# Lutein Intake and Blood Lutein Concentration Are Positively Associated with Physical Activity in Adults: A Systematic Review

**DOI:** 10.3390/nu10091186

**Published:** 2018-08-29

**Authors:** Madeline C. Cooke, Alison M. Coates, Elizabeth S. Buckley, Jonathan D. Buckley

**Affiliations:** School of Health Sciences, University of South Australia, Adelaide 5001, Australia; coomc006@mymail.unisa.edu.au (M.C.C.); alison.coates@unisa.edu.au (A.M.C.); elizabeth.buckley@unisa.edu.au (E.S.B.)

**Keywords:** exercise, carotenoids, behaviour, zeaxanthin

## Abstract

Lutein is a carotenoid that reduces the risk of some chronic diseases, possibly by altering physical activity behavior. The objective of this study was to conduct a systematic review of studies examining the relationship between lutein status (dietary intake/blood concentration) and physical activity. Peer-reviewed studies published in Medline, Web of Science, Cumulative Index to Nursing and Allied Health Literature (CINAHL), Scopus, and Embase were included if they reported a measure of association between lutein status and physical activity. Seventeen studies met the inclusion criteria. Eleven reported positive associations, three reported mixed results, and three reported no association. Two studies used objective measures of lutein status (blood concentration) and physical activity (accelerometry) and reported positive associations, with correlations of ≥0.36 and differences of ≥57% in physical activity between upper and lower tertiles. Studies using self-report measures reported weaker correlations (*r* = 0.06 to 0.25), but still more physical activity (18% to ≥600% higher) in those with the highest compared with the lowest lutein status. Higher lutein status may be associated with higher levels of physical activity, which may contribute to a reduced risk of chronic disease.

## 1. Introduction

Despite the health benefits of physical activity, many individuals are not physically active. In Australia, only ~55% of adults meet minimum physical activity guidelines of at least 150 min per week of moderate intensity physical activity [1]. The situation is similar in the USA, where national surveys report that only ~60% of adults meet national physical activity guidelines [2]. However, these surveys rely on self- or proxy-report data and, in the USA, an objective assessment of physical activity using accelerometry found that less than 5% of adults meet guidelines [2]. Physical inactivity is a major contributor to the burden of disease in Australia [3]. While many are unable to meet the minimum recommended physical activity guideline [4], recent data suggest that for sedentary individuals, even modest increases in physical activity can reduce disease risk [5].

Lutein is one of the most prevalent carotenoids in the human diet and is found in high levels in fruit and vegetables (1–60 mole %) [6]. A higher lutein status (dietary intake and/or blood lutein concentration) has been associated with a reduced risk of a range of chronic diseases, including cardiometabolic disease and some cancers [7]. The health benefits of lutein have been primarily ascribed to antioxidant, anti-mutagenic, and/or other effects on cell function [7]. However, physical activity is also associated with a reduced risk of these same chronic conditions [8] and preliminary evidence from randomised controlled trials (RCTs) in rats [9] and humans [10] showed that increasing circulating lutein concentrations through an increased intake was associated with increased physical activity. Matsumoto-Hagio et al. [9] added lutein to the chow of male juvenile rats housed in cages with running wheels for nine weeks. The lutein was added with or without a carrier (full fat milk), which facilitates lutein absorption. They found that blood lutein levels were only increased in the rats that consumed the chow with the lutein and milk carrier, and those rats increased their daily wheel running distance more than controls who consumed normal chow, chow with only the milk carrier added, or chow with just lutein added. Thomson et al. [10] performed an RCT in older sedentary humans, which demonstrated a statistically significant positive linear relationship between the magnitude of increase in plasma lutein concentration and the magnitude of increase in physical activity. Lutein is able to cross the blood–brain barrier and it has been hypothesized that it may influence brain centers that regulate physical activity [11]. Therefore, increasing dietary lutein intake might provide a means for increasing light physical activity in sedentary individuals.

Thus, preliminary evidence suggests that increasing lutein intake might increase physical activity. While additional RCTs are required to confirm the effect of lutein on physical activity, existing studies in the literature that have assessed lutein status (dietary intake and/or blood lutein concentrations) and physical activity may provide additional evidence to support continued research in this area. The aim of this review was to systematically evaluate the peer-reviewed literature to identify evidence of a relationship between lutein status and physical activity in adults. It was hypothesised that a higher lutein status would be associated with higher levels of physical activity.

## 2. Materials and Methods

In accordance with the Preferred Reporting Items for Systematic Reviews and Meta-Analyses (PRISMA) statement for improved reporting of systematic reviews [12], the protocol for this review was prospectively registered with PROSPERO (registration number: 42016046749) [13]. Peer reviewed publications of RCTs, cohort and cross sectional studies, or case series studies published in any year were included if they were performed in human adults; included a placebo or other control condition; and reported a quantifiable measure of dietary or blood lutein or lutein + zeaxanthin (an isomer of lutein), a quantifiable measure of physical activity, and a test of association between lutein status and physical activity. The primary outcome was the reported association between dietary intake or blood lutein/lutein + zeaxanthin concentration and physical activity. No limitations were set in relation to participants, interventions, comparisons, outcomes, or study design (PICOS).

A literature search was conducted on the 28th of September 2016. Databases searched were Medline, Embase, Scopus, Cumulative Index to Nursing and Allied Health Literature (CINAHL), and Web of Science. Two authors (M.C.C. and E.S.B.) conducted and replicated the search independently. The following search terms were used, including medical subject headings (MeSH) and text search terms. Example of search in the Medline database: [Adult (MeSH) or (adult* or elder* or senior* or geriatric*)] and [(lutein or zeaxanthin) or lutein (MeSH)] and [(level* or concentration*) adj5 (plasma or serum or blood or circulat*) or eating (MeSH) or eat* or ingest* or diet*)]. Included studies were limited to those in humans ≥17 years of age and reported in the English language. Reference lists of eligible studies identified were hand searched for additionally relevant studies.

Records of identified studies were stored in Endnote reference management software (Thomson Reuters, Philadelpia, Pennsylvania, USA, version X7.5.3) and duplicates were removed. The remaining references were uploaded to an online systematic review platform, Covidence© (www.covidence.org). The articles were screened independently and in duplicate by title and abstract in Covidence© (M.C.C all, A.M.C. and E.S.B. sharing second review, J.D.B. resolving conflicts). Studies were included at screening if they included a measure of lutein status and it was inferred that there may have been measures of physical activity, even when measurement of physical activity was not explicitly stated. Two separate reviewers then conducted the full-text eligibility assessment (M.C.C. screening all; J.D.B., A.M.C., and E.S.B. sharing second review). A third reviewer (J.D.B., A.M.C., or E.S.B.), who had not provided a second review of the full text article, was consulted if there was disagreement. Where there was duplication of data across multiple studies, only the original publication was included, unless subsequent publications provided additional data.

Data extraction and critical appraisal were performed in duplicate and independently (M.C.C. and either J.D.B., A.M.C., or E.S.B.). Conflicts were resolved in discussion with all authors. The Scottish International Guidelines Network (SIGN) randomized control trial critical appraisal tool (www.sign.ac.uk) was used to appraise randomized controlled trials and the Appraisal Tool for Cross-Sectional Studies (AXIS) [14] was used for cross sectional studies.

A descriptive analysis was used to synthesize the findings of included studies. Meta-analysis was not considered appropriate because of differences in study populations, large variability in the reported outcomes of lutein status and physical activity behavior, and a range of different measures being used to test the association between these outcomes.

## 3. Results

**Figure 1:**
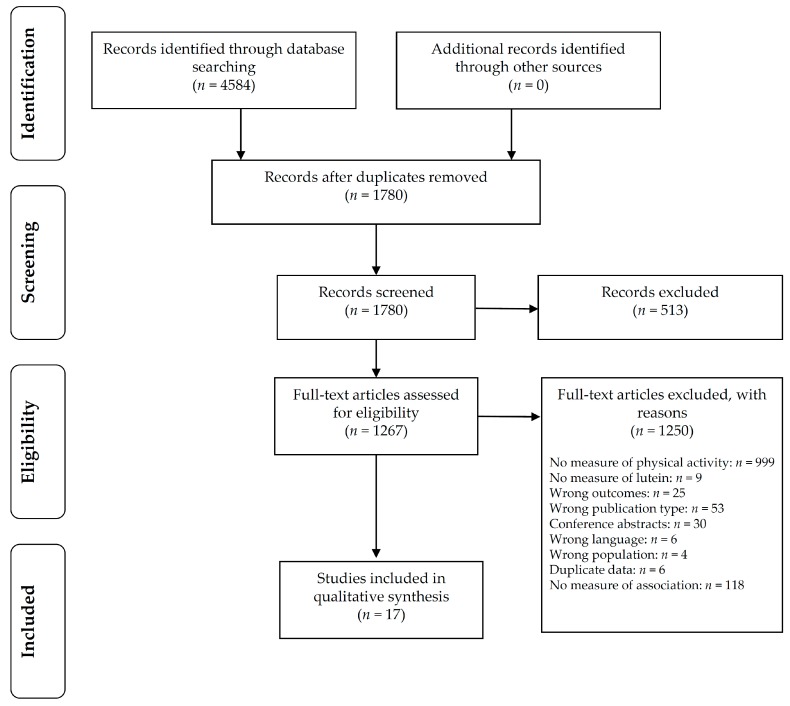
Literature search flow chart.

A total of 4584 articles with potentially relevant titles and abstracts were identified from searches. One thousand seven hundred and eighty articles remained after duplicates were removed. Five hundred and thirteen articles were excluded based on title and abstract, leaving 1267 that were collected as full text and assessed for inclusion. One hundred and thirty-five studies reported measures of both lutein status and physical activity, but of those, only 17 reported an association and were included (Figure 1). The majority (*n* = 16) of included studies were cross-sectional in design, and one was an RCT.

The majority of studies focused on middle-aged populations, with two smaller cross sectional studies looking specifically at younger adults [15,16]. One study reported exclusively on males [17], and two on females [18,19]. Of the nine studies reporting both sexes [15,20,21,22,23,24,25,26,27], two included predominantly female participants [23,26].

None of the eligible studies, other than the one RCT [10], had reported physical activity as a primary outcome. The primary outcomes for the remaining studies included relationships between lutein status and general demographics or lifestyle factors [17,18,23,28], serum y-glutamyl-transferase [15], diabetic retinopathy [29], neighborhood deprivation [25], age-related macular degeneration [30], metabolic syndrome [20], macular pigment optical density [21], fruit and vegetable intake [22], feelings of hostility [16], colon cancer [24], and cardiovascular disease [19,27].

Across eligible studies, physical activity was measured subjectively or objectively. Subjective measures of physical activity included self-report questionnaires [15,16,17,18,19,21,22,23,24,25,26,27,29,30] or responses to interviewer questions [28]. Objective measures were only reported by two studies [10,20], both of which used accelerometry.

Measures of lutein status were also either subjective or objective. Subjective measures included food records [18], food frequency questionnaires [21,23,28,29,30], or other dietary questionnaires [24,26]. Objective measures of lutein status were blood measures (serum or plasma). Three studies reported blood lutein alone [10,17,23] while eight reported the combination of blood lutein + zeaxanthin [15,16,19,20,21,22,25,27].

There was significant heterogeneity between reported measures of association for physical activity and lutein status (see Table 1). However, of the 17 eligible studies, 11 reported positive associations between lutein status and physical activity [10,15,16,19,20,23,25,26,27,28,30], three reported mixed results [17,21,24], and three reported no association [18,22,29]. No studies reported a negative association.

### 3.1. Synthesis of Results

The highest level of evidence, based on Australian National Health and Medical Research Council (NHMRC) guidelines for ranking of intervention study designs [31], was the RCT that evaluated whether dietary supplementation with lutein compared with placebo for four weeks influenced physical activity and/or sedentary behavior in older men and women (55–80 years, *n* = 36) [10]. Plasma lutein concentrations increased by 135% in participants who consumed the lutein supplements, and their physical activity (daily activity counts from accelerometry) increased by 18% more than controls, although this did not reach statistical significance (*p* = 0.08). Similarly, compared with controls, there was a tendency for a reduction in time spent sedentary (9% reduction, i.e., 20 min per day) in participants who consumed lutein, but this did not reach statistical significance (*p* = 0.14). This is most likely due to the study being underpowered because of a small sample size (*n* = 36). However, while there were no significant overall differences in changes in physical activity or sedentariness, after controlling for total cholesterol, the percentage change in plasma lutein concentration was positively correlated with the percentage change in physical activity accelerometry counts (*r* = 0.36, *p* = 0.03) and inversely correlated with percentage changes in time spent sedentary (*r* = −0.39, *p* = 0.02). This RCT was one of the few eligible studies that reported objective measures of both lutein status (plasma lutein concentration) and physical activity (accelerometry). The only other eligible study to report objective measures of lutein status (serum lutein + zeaxanthin) and physical activity (step counts by accelerometry) was a cross-sectional analysis of the 2005–2006 National Health and Nutrition Examination Survey (NHANES) (*n* = 1930) by Choi et al. [20]. This study also reported a positive relationship between serum lutein/zeaxanthin concentrations and increasing tertiles of step counts in both males and females (*p* < 0.01) when adjusting for age, body mass index, and total energy intake. The highest tertile of physical activity had ~26% higher serum lutein + zeaxanthin concentrations (18.6 ug/dL vs. 14.8 ug/dL) and performed at least 57% more steps per day compared with the lowest tertile. 

Kitamura et al. [17] evaluated associations between lifestyle factors, including physical activity assessed by a lifestyle questionnaire, and serum lutein concentration in Japanese men aged 24–60 years who smoked >15 cigarettes per day. The authors found no significant relationship between serum lutein concentration and daily walking time (*r* = 0.01, *p* > 0.05), but serum lutein was positively related to frequency of participation in sport when adjusted for age (*r* = 0.12, *p* < 0.05). Ciulla et al. [21] reported on both subjective and objective measures of lutein status in 280 men and women aged 18–50 years who were recruited by advertisement into a study evaluating nutrition and eye health. Dietary intake of lutein + zeaxanthin was assessed by food frequency questionnaire (FFQ) and serum measures of lutein + zeaxanthin were also assessed. The authors adjusted for a large number of covariates that might have influenced lutein status in their analysis, including tobacco use, gender, age, and body mass index. The number of times per week that exercise was performed was assessed using a questionnaire. The authors found no significant association between the frequency of participation in exercise and serum lutein + zeaxanthin (*r* = 0.02, *p* > 0.05), but there was a significant association with dietary lutein + zeaxanthin intake (*r* = 0.25, *p* < 0.05) [21].

A number of other cross sectional studies [15,16,19,25,28] reported positive relationships between blood lutein and zeaxanthin concentrations and self-report measures of physical activity. Lee et al. [15] used data from 3128 men and women aged 17–35 years who were enrolled in the Coronary Artery Risk Development in Young Adults (CARDIA) study to evaluate the association of serum carotenoids and tocopherols with future risk of elevated serum γ-glutamyltransferase, a marker of liver disease and cardiovascular disease risk. Serum carotenoid concentrations were measured at the same time that physical activity was assessed using the CARDIA Physical Activity History questionnaire, which assesses sport, exercise, leisure, and occupational physical activity over the previous 12 months. After adjusting for race, sex, age, alcohol consumption, body mass index, smoking status, low-density lipoprotein (LDL)-cholesterol, high-density lipoprotein (HDL)-cholesterol, and trigylcerides, there was a significant correlation between serum lutein + zeaxanthin and total physical activity score (*r* = 0.08, *p* < 0.01), indicating that higher serum lutein + zeaxanthin was associated with more physical activity. In another analysis from the CARDIA study, but using a different sample (*n* = 3579 men and women aged 18–30 years), Ohira et al. [16] found a significant correlation between serum lutein + zeaxanthin and total physical activity score when adjusted for age, gender, race, and serum lipid concentrations (*r* = 0.06, *p* < 0.01). Similarly, a study by Gruber et al. [28], which comprised a cross-sectional analysis of 7059 participants (≥40 years of age) from the 1988–1994 NHANES survey, reported that physical activity status (by interview) was directly related to serum lutein/zeaxanthin concentrations and that participants who were physically active had serum levels that were 13% higher than those who were not physically active in an unadjusted analysis (*p* < 0.01). A larger cross-sectional study (*n* = 17,002) by Stimpson et al. [25] also used data from the third NHANES survey (1988–1994), but included younger participants (≥17 years of age) than in the study by Gruber et al. [28] and, while Gruber et al. had defined physical inactivity as falling within the lower two quartiles of physical activity, Stimpson et al. defined physical inactivity as not participating in any physical activities in the previous month. After controlling for age, sex, race, years of education, household income, employment status, smoking status, alcohol use, body mass index, and total cholesterol, Stimpson et al. [25] reported a significant negative association between no engagement in physical activity in the past month and combined serum lutein and zeaxanthin concentrations (ß = −1.10, *p* < 0.001), indicating that higher serum lutein + zeaxanthin concentrations were associated with performing more physical activity. Similarly, Wang et al. [19] evaluated associations between plasma carotenoids and risk factors for cardiovascular disease, including physical activity in 2895 older women (≥45 years) using data from the Women’s Health Study. In an unadjusted analysis, the authors reported that compared with women who rarely or never exercised, there was no difference in serum lutein + zeaxanthin concentration for women who reported exercising less than once per week, but those who reported exercising 1–3 times per week or ≥4 times per week had significantly higher serum lutein + zeaxanthin concentrations (*p* < 0.001).

Five studies reporting subjective measures of lutein status (dietary intake) and physical activity reported positive associations [23,24,26,27,30]. Moeller et al. evaluated data from 1787 women aged 50–79 years with low (below 28th percentile) or high (above 78th percentile) lutein + zeaxanthin intakes (assessed using a semi-quantitative food frequency questionnaire) in the Carotenoids in Age-Related Eye Disease (CAREDS) study [30]. After adjusting for age, they found that participants with high dietary lutein + zeaxanthin intakes performed 50% more physical activity per week compared with those with low dietary intakes (12 metabolic equivalents (METs)/week vs. 18 METs/week, *p* ≤ 0.001). Rock et al. [23] evaluated the relationship between dietary lutein + zeaxanthin intake and physical activity assessed by interviewer led questionnaires in 2786 participants aged 18–92 years. When controlling for age, race, education level, sex, body mass index, smoking status, alcohol consumption, and dietary energy intake, it was found that participants who engaged in 30–60 min of physical activity per day had a 10.3% higher dietary lutein intake compared with those who engaged in less than 30 min per day (*p* < 0.05). Those who engaged >60 min per day had 19.3% higher dietary lutein intake (*p* < 0.05). This was suggestive of a dose-response effect of dietary lutein intake on physical activity. The other two studies, using only subjective measures for both lutein status and physical activity, also showed a positive relationship. A case-control study of colon cancer by Slattery & Potter [24], with 1993 cases and 2410 controls, after adjusting for age, found a weak significant positive association between lutein intake and long-term vigorous physical activity (*r* = 0.08, *p* < 0.05) for male participants (*n* = 1290), with females (*n* = 1120) showing a slightly weaker association that was not statistically significant (*r* = 0.05, *p* > 0.05). The correlation analysis included both cases and controls in the same analysis. Similarly, Tormo et al. evaluated patterns of nutrient intake according to levels of sport physical activity in 37,287 participants enrolled in the European Prospective Investigation on Cancer (EPIC) study [26]. There was a linear trend (*p* ≤ 0.05) for increasing dietary lutein intake across increasing levels of physical activity. Participants engaging in 0–0.5 h per week of sport activity were used as the reference and compared with those engaging in >0.5–2 h/week, >2–3 h/week, and >3 h/week. Dietary lutein intake was higher for all levels of sport activity compared with the reference (*p* ≤ 0.05), and this was maintained when the analysis was adjusted for age, body mass index (BMI), current smoking, alcohol consumption, higher education, sedentariness at work, and interaction of education with physical activity at work (*p* ≤ 0.05), suggesting an independent relationship between increasing dietary lutein intake and increasing sport activity engagement. Wang et al. [27] undertook a cross-sectional analysis of 2003–2006 NHANES data to evaluate associations between dietary carotenoid intakes and cardiovascular disease risk, and whether serum carotenoid concentrations mediated the strength of any associations. Dietary intake of carotenoids (by repeat 24 h recall), serum carotenoid concentrations, and physical activity (by interview) were measured, but only the association between physical activity and dietary carotenoid intake was tested. In their analyses, the authors adjusted for age, gender, ethnicity, income, supplement use, alcohol use, smoking status, diabetes status, cholesterol, triglycerides, prescription medication use, and dietary energy intake. This analysis indicated that there was a higher combined dietary lutein + zeaxanthin intake in individuals who engaged in 2.5 to <11.5 MET-h/week of physical activity per week, compared with those who were sedentary (<2.5 MET-h/week; *p* < 0.017) [27]. However, dietary lutein/zeaxanthin intake did not differ between participants who engaged in >11.5 MET-h/week of activity and those who were sedentary, suggesting an inverted U-shaped relationship between dietary lutein/zeaxanthin intake and physical activity.

Three studies found no significant relationship between lutein status and physical activity [18,22,29]. Hamulka et al. [18] sought to assess the influence of selected demographic and lifestyle factors on dietary lutein intake in a small sample (*n* = 100) of randomly selected Polish women aged 19–81 years. Dietary food records including three week days and one weekend day were used to assess dietary intake, with lutein intake being estimated based on the lutein content of foods in the Polish market that had been established in an earlier study [32]. Physical activity was assessed by questionnaire. There were no significant differences in dietary lutein intake between participants who were sedentary, or those who engaged in moderate or high levels of physical activity (*p* = 0.33), and there was no significant relationship between dietary lutein intake and physical activity (*r* = −0.105, *p* = 0.30) even when adjusted for age, BMI, place of dwelling, and education level (*r* = −0.062, *p* = 0.55). Coyne et al. [22] used data from 1598 adults aged ≥25 years to evaluate serum carotenoid and folate concentrations to evaluate responses to self-administered brief questionnaires regarding consumption of fruit and vegetables. This study was a sub-analysis of a larger study that aimed to determine the prevalence of diabetes and associated risk factors, including physical activity assessed by questionnaire. Analyses were adjusted for age, sex, vitamin use, body mass index, smoking status, alcohol intake, LDL-cholesterol, HDL-cholesterol, total cholesterol, and triglycerides. There was no significant difference in serum lutein + zeaxanthin between participants that were sedentary (no participation in physical activity in the last week) and those who were insufficiently active but not sedentary (<150 min of physical activity in the last week) or those who were sufficiently active (>150 min of physical activity in the last week). Sahli et al. [29] evaluated data from 1430 participants with diabetes from the Atherosclerosis Risk In Communities (ARIC) study [33] in an effort to identify whether the dietary intake of lutein was associated with the prevalence of diabetic retinopathy. Dietary intake was assessed using a food frequency questionnaire and physical activity was assessed using an interviewer-administered Modified Baecke Physical Activity questionnaire. After adjusting for dietary energy intake, no differences were found in work, sport, or other leisure time physical activity between participants in the highest and lowest quartiles of dietary lutein intake (*p* > 0.25).

### 3.2. Risk of Bias within Studies

The randomized control trial [10] had acceptable control for risk of bias, with the only concern being a small difference in body weight (2.8 kg) between control and intervention groups at baseline. There was some variability in risk of bias scores across the cross-sectional studies, particularly in relation to justification of sample size [16,19,21,23,24,26,27,29], categorization of non-responders [18,23,29,30], and internal consistency of results [16,19,22,25,26,27,28,30]. Justification of sample size was difficult to determine for some studies as most did not report an a priori power analysis, but assumed that a large sample size reflected an adequate sample size [16,19,23,24,26,27,29]. Internal consistency of outcomes of interest was not reported by most cross-sectional studies. However, this might be because of associations between lutein status and physical activity not generally being the primary interest of the study.

Most studies used subjective self-report measures for lutein status (i.e., dietary intake) and physical activity. Both self-reported dietary intake of carotenoids [34] and physical activity [2] are subject to reporting bias, which might have influenced some of the reported associations. Only two studies [10,20] used objective measures of both physical activity (accelerometry) and lutein status (blood concentrations), but both reported positive associations between the two measures. In addition, in most studies, the associations between lutein status and physical activity were assessed as secondary outcomes and as such, potential confounders of this relationship were rarely controlled for, thus making it difficult to determine the strength of any independent relationship between the two.

## 4. Discussion

The main finding of this review was that the majority of studies that assessed relationships between lutein status and physical activity reported positive associations, such that a higher lutein status was associated with more physical activity. Most studies that reported a positive association were cross-sectional, with only one being an RCT [10], thus a causative effect of lutein status on physical activity cannot be implied.

The hypothesis that lutein might increase physical activity is novel. Therefore, while 135 studies were identified that had measured both lutein status and physical activity, only 17 of them formally tested whether there was a relationship between the two. Of those that did formally test the relationship, only one was specifically interested in whether a higher lutein status was associated with higher levels of physical activity, and that was an RCT performed by our team [10]. In other studies, the association was a secondary analysis, and in only one of those studies was it specifically discussed. Gruber et al. [28], who sought to identify lifestyle determinants of lutein status because of the benefits of a high lutein status for eye health, found that higher serum lutein + zeaxanthin concentrations were positively associated with higher levels of physical activity, and interpreted this finding as indicating that physical activity might increase lutein status, but indicated that a mechanism by which this might occur had not been identified. Kitamura et al. [17] evaluated determinants of serum carotenoids and other micronutrients associated with anticarcinogenic and anti-atherosclerotic effects in Japanese men aged 24–60 years who smoked >15 cigarettes per day. They identified a positive association between sport participation and serum lutein concentration, as well as serum concentrations of cryptoxanthin, retinol, and alpha-tocopherol. They did not specifically discuss the relationship between dietary lutein intake and sport participation, but indicated that they had no plausible explanation for any of the relationships. The lack of formal assessment and/or discussion of associations between lutein status and physical activity in studies that had measured relevant outcomes is indicative of the novelty of the hypothesis that lutein status might influence physical activity.

While most studies eligible to be included in this review identified a positive association between lutein status and physical activity, the majority were cross-sectional. Therefore, because fruit and vegetables are the primary dietary source of lutein, it could be argued that people who consume more fruit and vegetables, and thus have a higher blood lutein status, might be more health conscious and also choose to be more physically active. A number of studies have shown that people who are more physically active consider it more important to eat nutritious foods [35] and consume healthier diets [11,36,37]. However, the preliminary evidence from the trial in rats [9] and the double-blind RCT in humans [10] suggests that lutein status influences physical activity.

A high lutein status is associated with a reduced risk of a range of chronic diseases, with the risk reduction having been primarily attributed to antioxidant, anti-mutagenic, and/or other effects of lutein on cell function [7]. Increasing physical activity also reduces the risk of developing a range of chronic diseases [8]. Evidence from this review suggests that at least part of the effect of lutein on reducing the risk of chronic disease might be due to it increasing physical activity. The only study that categorized the intensity of the additional physical activity associated with an increased lutein status was the RCT by Thomson et al. [10], which indicated that physical activity increased primarily because of an increase in light activity. Recent evidence suggests that increasing light activity can have health benefits for individuals who are sedentary. Schmid et al. [38] evaluated data from 3702 adults from NHANES (2003–2006) who had physical activity assessed at baseline by accelerometry and were followed prospectively for mortality over 6.4 years. Using temporal modelling, they found that replacing 30 min of sedentary time with light physical activity in sedentary adults reduced mortality by 14%. Similarly, Fishman et al. [39] followed 3029 adults from NHANES (2003–2006) for five years and found that replacing 30 min of sedentary time with light physical activity was associated with a 20% reduction in mortality risk. In the present review, the differences in physical activity between lowest and highest lutein status in the studies identified ranged from 18–600% and, based on data from Thomson et al. [10], we speculate that the greater physical activity was of light intensity. Self-report data indicates that on average, Australian adults spend just over 30 min per day doing physical activity [40]; therefore, an increase in physical activity of 18–600% would represent an increase of between five minutes and three hours. Based on a 14–20% reduction in mortality risk when half an hour of sedentariness is replaced by light activity, this would equate to a reduction in mortality risk of 2.3% to >84%. While a 2.3% reduction in mortality risk is not large, given the prevalence of inactivity, when applied to such a large proportion of the population that is sedentary, this would potentially have important implications for population health and health care systems.

The mechanism by which lutein might increase physical activity is not clear, but lutein crosses the blood–brain barrier and accumulates in brain regions involved in behavioral regulation, including the frontal cortex [41]. Lutein has been suggested to modulate functional properties of neurons and influence inter-neuronal communication [42]. This has been proposed as a mechanism to explain effects of lutein on cognitive function that have been observed in some studies [42], but might also contribute to changes in behavior, including alterations in physical activity. Additional studies should seek to evaluate relationships between macular optical pigment density (a biomarker of lutein/zeaxanthin that has crossed the blood brain barrier) and physical activity, and RCTs should evaluate whether there is a causative effect of an increased lutein intake on physical activity.

A number of limitations affect the interpretation of outcomes of this systematic review. The majority of eligible studies assessed dietary lutein intake and physical activity using self-report measures (questionnaires or interviews), which are subject to reporting bias [2,34]. Most cross-sectional studies controlled for factors that might influence lutein status, and also possibly the relationship between lutein status and physical activity, such as age, smoking status, and alcohol intake, amongst others, but there may have been some residual confounding, and these should be stratified in future RCTs to guard against confounding. Also, some studies did not separately report lutein and zeaxanthin measures, but included a combined measure of both (i.e., lutein + zeaxanthin), making it difficult to discern whether associations were due to lutein, zeaxanthin, or both. However, Thomson et al. [10] used supplements containing both lutein and zeaxanthin and found an almost-significant inverse relationship between increases in plasma zeaxanthin and reductions in sedentary time (*r* = −0.30, *p* = 0.07), suggesting that zeaxanthin, which is an isomer of lutein, might also play a role in mediating physical activity behavior, but the study was underpowered to detect this relationship as being statistically significant. A strength of this systematic review was that there was minimal risk of publication bias across eligible studies, as the hypothesis that lutein might be associated with increased physical activity is so novel that all but one study did not report the association between lutein status and physical activity as the primary outcome.

## 5. Conclusions

In conclusion, this systematic review provides evidence of a positive relationship between lutein status (dietary intake and/or blood lutein concentrations) and physical activity. If increasing lutein status, or possibly also the status of other carotenoids, is able to increase physical activity, this might be useful for improving physical activity to mitigate the risk of chronic disease. However, large-scale RCTs are required to confirm effects on physical activity and any associated health benefits.

## Figures and Tables

**Table 1:** Summary of included studies.

Reference	Study Design	NHMRC Level of Evidence	CA Score	Sample Characteristics	Physical Activity Outcome Measure	Physical Activity Measure	Lutein Outcome Measure	Measure of Lutein Status	Measure of Association (Type, Strength, Direction, and Significance)
*Randomised control trials*									
Thomson et al. (2014) [10]	RCT	II	7	Australia *n* = 44 Sedentary, but otherwise healthy males and females 68.8 ± 6.4 years BMI 25.3 ± 2.6 kg/m^2^	Subjective and objective Amount of sedentary PA, light PA and mod–vig PA by exercise diary Cut-point PA levels using accelerometer (7164 ActiGraph) over 7-day period	Lutein (Mean values) Sed: 235 min/day (SD 61), light PA: 301 min/day (SD 88), mod-vig PA: 22 min/day (SD 14) Accelerometer 235,292 counts per day (SD 82, 693) Placebo Sed: 219 mins/day (SD 46), light PA: 341 min/day (SD 76), mod-vig PA: 24 min/day (SD 18) Accelerometer 273,760 counts per day (SD 85, 018)	Objective HPLC (plasma lutein and zeaxanthin separately)	Lutein: mean 10.3 ug/dL (SD 2.5) Placebo: mean 10.1 ug/dL (SD 3.6)	(1) Correlation SEDENTARY: Plasma lutein and time spent sedentary: *r* = −0.36 (*p* = 0.03) PA: Plasma lutein and activity counts: *r* = 0.29, *p* = 0.08 (2) % change correlation SEDENTARY: Time sedentary and relative % change in plasma lutein: *r* = −0.39 (*p* = 0.02) PA: % difference lutein with % difference activity, *r* = 0.36, *p* = 0.03
*Cross sectional studies ^*									
Choi et al. (2016) [20]	Cross sectional	IV	17	USA *n* = 1661 NHANES 40–70 years	Objective Accelerometer daily steps (sedentary [s], intermediate [i], and active [a])	Male: s <6802 steps/day i = 6082–10, 698 steps/day a >10, 698 steps/day Female: s < 5785 steps/day i = 5785–9225 steps/day a ≥ 9226 steps/day	Objective Serum lutein and zeaxanthin	Male: s = 14.0 ug/dL mean (SE 0.7) i = 16.3 ug/dL mean (SE 0.6) a = 18.9 ug/dL mean (SE 0.6) Female: s = 14.8 ug/dL mean (SE 0.6) i = 17.1 ug/dL mean (SE 0.5) a = 18.6 ug/dL mean (SE 0.5)	ANCOVA tertiles of step counts. Covariates: age, BMI, total energy intake M: positive increase serum lutein/zeaxanthin with increasing tertile step counts (*p* < 0.01) F: positive increase serum lutein/zeaxanthin with increasing tertile step counts (*p* < 0.01) >26% higher serum lutein + zexanthin in active vs sedentary participants Associated with >57% difference in accelerometer step counts between sedentary and active participants
Coyne et al. (2005) [22]	Cross sectional	IV	17	Australia *n* = 1598 Random sample of adults ≥25 years	Subjective Self-report questionnaire previous week. PA time: sum of time walking or moderate intensity activity plus double time vigorous activity	Sufficiently active >150 min/week In-sufficiently active <150 min/week Sedentary 0 min/week	Objective HPLC serum lutein/zeaxanthin	Active: 0.39 umol/L mean (95% CI 0.37–0.41) In-sufficiently active: 0.39 umol/L mean. (95% CI 0.37–0.41) Sedentary: 0.37 umol/L mean (95% CI 0.34–0.39)	ANOVA No significant difference serum lutein/zeaxanthin across PA tertiles, *p* > 0.01
Gruber et al. (2004) [28]	Cross sectional	IV	15	USA *n* = 7059 NHANES ≥40 years	Subjective Interview	PA ‘yes’ by quintiles of serum lutein/zeaxanthin Q1: 51%, Q3: 62%, Q5: 69%.	Objective HPLC serum lutein/zeaxanthin	Q1: 0.02–0.25 umol/L Q3: 0.33–0.44 umol/L Q5: 0.58–4.45 umol/L	Participants who were physically active had 13% higher serum lutein/zeaxanthin than those who were not active, *p* < 0.01
Kitamura et al. (1997) [17]	Cross sectional	IV	15	Japan *n* = 194 Healthy male smokers (>15 cigarettes/day) 24–60 years	Subjective Self-reported questionnaire (closed questions)	Average duration walking/day (<30 min, 30 min to 1 h, 1–2 h, >2 h) Frequency participating in sports (none, occasional, frequent)	Objective HPLC (serum lutein)	HPLC: Mean 39.2 ug/dL (95% CI 37.5–41.0)	Spearman rank correlation coefficient (adjusted for age) Between walking time and serum lutein, *r* = 0.01 (*p* > 0.05) Between frequency of sport participation and serum lutein, *r* = 0.12 (*p* < 0.05)
Lee et al. (2004) [15]	Cross sectional	IV	15	USA *n* = 3128 Black and white males and females 17–35 years	Subjective CARDIA PA history, Minnesota Leisure time PA questionnaire (Simplified version)	Data not reported	Objective HPLC serum lutein/zeaxanthin	Data not reported	Linear regression analysis *r* = 0.08 (*p* < 0.01)
Slattery & Potter (2002) [24]	Cross sectional (case control)	IV	15	USA Colon cancer cases *n* = 1993 Control *n* = 2410 30–79 years	Subjective CARDIA PA questionnaire (Scoring 1 = no vigorous leisure time PA) 2 = 1–250 cal/week. 3 = 251–1000 cal/week. 4 ≥ 1000 cal/week)	Men: Case: 1: *n* = 233, 2: *n* = 312, 3: *n* = 329, 4: *n* = 225. Control: 1: *n* = 216, 2: *n* = 314, 3: *n* = 379, 4: *n* = 380 Women: Case: 1: *n* = 326, 2: *n* = 233, 3: *n* = 189, 4: *n* = 146. Control: 1: *n* = 318, 2: *n* = 314, 3: *n* = 264, 4: *n* = 224	Subjective Nutrient values calculated using Minnesota NCC database	Data not reported	(1) Correlation coefficient: Male: *r* = 0.08 (*p* < 0.05) Female: *r* = 0.05 (*p* > 0.05)
Hamulka et al. (2009) [18]	Cross sectional	IV	14	Poland *n* = 100 Female 48.6 ± 16.2 years BMI 24.6 kg/m^2^	Subjective Self-reported questionnaire	Sedentary, moderate, high (values not reported)	Subjective Dietary lutein intake from food records	Sed: mean 2.02 mg/day (SD 0.67) Mod: mean 2.29 mg/day (SD 1.21) High: mean 1.85 mg/day (SD 0.74)	Non-parametric Kruskal–Wallis ANOVA, *p* = 0.33 Spearman rank correlation: Crude: *r* = 0.105 (*p* = 0.30) Adjusted for age, BMI, place of dwelling and level of education: *r* = −0.062 (*p* = 0.55)
Sahli et al. (2016) [29]	Cross sectional	IV	14	USA *n* = 1430 Black and white male and female 45–65 years Diabetes mellitus	Subjective Modified Baecke PA questionnaire	*By quintiles of lutein intake* Q1: PA at work 2.2 (SD 0.6) Sports in leisure time 2.3 (SD 0.8) Other leisure time PA 2.3 (SD 0.6) Q4: PA at work 2.1 (SD 1.0) Sports in leisure time 2.4 (SD 0.7) Other leisure time physical activity 2.3 (SD 0.6)	Subjective (outcome measure not reported)	Q1: mean 435.2 ug/1000 kcal (SD 165.1) Q4: mean 4853.1 ug/1000 kcal (SD 2695.3)	ANOVA PA at work index *p* = 0.29 Sports in leisure time index *p* = 0.89 Other leisure time PA index *p* = 0.25
Stimpson et al. (2007) [25]	Cross sectional	IV	13	USA *n* = 17, 002 NHANES ≥17 years	Subjective Self-reported questionnaire	PA score of 0: *n* = 11,757 PA score >1: *n* = 5236 Missing: *n* = 9	Objective HPLC serum lutein/zeaxanthin	PA score 0: mean 22.62 ug/dL (SD 12.59) PA score ≥1: mean 23.17 ug/dL (SD 13.33)	Multivariate linear regression ≥1, using high PA as a reference B value = −1.10 (SE 0.34) *p* < 0.01. Indicating that as serum lutein + zeaxanthin increased physical inactivity decreased
Tormo et al. (2003) [26]	Cross sectional	IV	13	Spain *n* = 37, 287 Healthy male and female 50.9 ± 7.2 years BMI 28.4 ± 3.4 kg/m^2^ 31% smokers 17% heavy drinkers	Subjective PA questionnaire	0–0.5 h/week, >0.5–2 h/week, >2–3 h/week, >3 h/week	Subjective Lutein intake, food recalls against Food Composition table	0–0.5 h/week (ref): mean 784.7 ug/day (SD 826.0) >0.5–2 h/week mean 898.8 ug/day (SD 828.6) >2–3 h/week mean 935.6 (SD 910.7) >3 h/week mean 854.7 ug/day (SD 840.0)	*p* < 0.05 for ANOVA comparing mean value of each PA category with reference level *p* < 0.05 for ANCOVA comparing mean PA category with reference level adjusted by age, BMI, current smoking, excessive alcohol drinking, secondary/higher education, sedentary PA at work and interaction of education with PA at work ≥600% difference in hours of PA
Ciulla et al. (2001) [21]	Cross sectional	IV	12	USA *n* = 280 Male and female 18–50 years 26% smokers	Subjective Self-reported questionnaire	Number of times exercise per week	Objective Serum lutein/zeaxanthin (umol/L) Subjective FFQ lutein/zeaxanthin (ug/day)	Serum: 0.372 umol/L mean (SD 0.169) Intake: 1102 ug/day mean (SD 839)	Spearman correlation coefficient Serum: *r* = 0.02, *p* > 0.05 Intake: *r* = 0.25, *p* < 0.05
Moeller et al. (2006) [30]	Cross sectional	IV	12	USA *n* = 1787 Female 50–79 years	Subjective Self-reported questionnaire	Physical activity levels Low: 12 MET/week High PA: 18 MET/week	Objective HPLC serum lutein/zeaxanthin Subjective Dietary intake questionnaire	Dietary lutein intake Low: mean 792 ug/day (SD 169) High: mean 2868 ug/day (SD 919)	No measure of association between serum lutein and physical activity reported 50% difference in physical activity between low and high dietary intake groups (*t*-test, *p* ≤ 0.001).
Ohira et al. (2008) [16]	Cross sectional	IV	12	USA *n* = 3579 18–30 years	Subjective Self-reported total PA score, habitual PA, and participation in 13 different PA categories (vigorous to moderate) over 12 months	Total CARDIA PA history score (arbitrary units)	Objective HPLC 12-h fasting serum lutein/zeaxanthin	Data not reported	Correlation coefficient, adjusted for age, gender, race, and serum lipid *r* = 0.06, *p* < 0.01
Wang et al. (2008) [19]	Cross sectional	IV	11	USA *n* = 2895 Female ≥45 years Self-reported free from cardiovascular disease and cancer (except non-melanoma skin cancer)	Subjective Questionnaire: self-reported vigorous PA	Rarely/never (ref), <1 time/week, 1–3 times/week, >4 times/week	Objective HPLC serum lutein/zeaxanthin	Reported as mean (95% CI) Rarely/never (ref): 0.279 umol/L (0.271–0.286) <1 time/week: 0.284 umol/L (0.274–0.296) 1–3 times/week 0.300 umol/L (0.290–0.309) >4-times/week: 0.310 umol/L (0.293–0.328)	Serum lutein + zeaxanthin significantly higher in 1–3 times/week and >4 times/week compared with rarely or never (*t*-test) *p* < 0.001 >400% difference between reference and >4 times per week of physical activity
Rock et al. (2002) [23]	Cross sectional	IV	9	USA *n* = 2786 Male and female 44 ± 16 years BMI 27.5 ± 6.1 kg/m^2^	Subjective Questionnaire, PA minutes/day	<30 min/day (ref), 30–60 min/day or >60 min/day	Subjective Dietary lutein/zeaxanthin intake	Mean intake 1347 (891) ug/day	% difference in dietary intake of lutein + zeaxanthin from reference physical activity group (<30 min/day) 30–60 min/day: 10.3% higher dietary intake (5.6–15.3%) *p* = 0.05 >60 min/day: 19.3% higher dietary intake (9.6–29.8%) *p* = 0.05
Wang et al. (2014) [27]	Cross sectional	IV	9	USA *n* = 2856 NHANES Male and female ≥ 20 years	Subjective Questionnaire	<2.5 MET h/week (ref) 2.5–<4 MET h/week 4–<11.5 MET h/week >11.5 MET h/week	Subjective Dietary lutein/zeaxanthin intake	<2.5 MET h/week: dietary intake of 0.65 mg/day (0.59–0.71) 2.5–<4 MET h/week: dietary intake of 0.84 mg/day (0.75–0.94) 4–<11.5 MET h/week: dietary intake of 0.82 mg/day (0.73-0.92) >11.5 MET h/week: dietary intake of 0.75 mg/day (0.68–0.83)	Multivariate model geometric means (95% CIs) Dietary lutein/zeaxanthin intake for 2.5–<4 MET h/week and 4–<11.5 MET h/week significantly different from <2.5 MET-h/week (ref) (*p* < 0.017) >400% difference in physical activity between dietary lutein intake of 0.65 mg/day (<2.5 MET h/week group) and dietary intake of 0.82–0.84 mg/day (2.5 to <11.5 MET h/week groups)

^ Cross sectional studies are presented in descending order from highest to lowest critical appraisal score, then alphabetically by first author, and then by most recent publication year. CA score: critical appraisal score, RCT: randomised controlled trial, FFQ: food frequency questionnaire, HPLC: high performance liquid chromatography, CI: confidence interval, *r*: correlation coefficient, OR: odds ratio, SD: standard deviation, SE: standard error, NHANES: National Health and Nutrition Examination Survey (USA), NCC: Nutrition coordinating center, PA: physical activity, con: control, sed: sedentary, mod: moderate, ref: reference, ANOVA: analysis of variance, BMI: body mass index, ANCOVA: analysis of covariance, MET: metabolic equivalent, ARIC: the atherosclerosis risk in communities study, Q: quintile.
